# The broad pathogenetic role of *TCF7L2* in human diseases beyond type 2 diabetes

**DOI:** 10.1002/jcp.30581

**Published:** 2021-10-06

**Authors:** Laura del Bosque‐Plata, Eduardo Pavel Hernández‐Cortés, Claudia Gragnoli

**Affiliations:** ^1^ Laboratorio de Nutrigenética y Nutrigenómica Instituto Nacional de Medicina Genómica Mexico City Mexico; ^2^ Department of Medicine, Division of Endocrinology, Diabetes, and Metabolic Disease, Sidney Kimmel Medical College Thomas Jefferson University Philadelphia Pennsylvania; ^3^ Division of Endocrinology Creighton University School of Medicine Omaha Nebraska USA; ^4^ Department of Public Health Sciences Penn State College of Medicine Hershey Pennsylvania USA; ^5^ Molecular Biology Laboratory Bios Biotech Multi‐Diagnostic Health Center Rome Italy

**Keywords:** cancer, Crohn's disease, diabetes, *TCF7L2* gene, Wnt‐signaling pathway

## Abstract

The TCF7L2 protein is a key transcriptional effector of the Wnt/β‐catenin signaling pathway, regulating gene expression. It was initially identified in cancer research and embryologic developmental studies. Later, the *TCF7L2* gene was linked to type 2 diabetes (T2D), implicating *TCF7L2* and Wnt‐signaling in metabolic disorders and homeostasis. In fact, *TCF7L2*‐T2D variants confer the greatest relative risk for T2D, unquestionably predicting conversion to T2D in individuals with impaired glucose tolerance. We aim to describe the relevance of *TCF7L2* in other human disorders. The *TCF7L2*‐single nucleotide polymorphisms (SNPs) and T2D‐risk association have been replicated in numerous follow‐up studies, and research has now been performed in several other diseases. In this article, we discuss common *TCF7L2*‐T2D variants within the framework of their association with human diseases. The *TCF7L2* functional regions need to be further investigated because the molecular and cellular mechanisms through which *TCF7L2* contributes to risk associations with different diseases are still not fully elucidated. In this review, we show the association of common *TCF7L2*‐T2D variants with many types of diseases. However, the role of rare genetic variations in the *TCF7L2* gene in distinct diseases and ethnic groups has not been explored, and understanding their impact on specific phenotypes will be of clinical relevance. This offers an excellent opportunity to gain a clearer picture of the role that the *TCF7L2* gene plays in the pathophysiology of human diseases. The potential pleiotropic role of *TCF7L2* may underlie a possible pathway for comorbidity in human disorders.

## 1 INTRODUCTION

The transcription factor 7‐like 2 (*TCF7L2* gene), previously named transcription factor 4 (TCF4)—not to be mistaken for TCF4, associated with a range of neuropsychiatric phenotypes, including autism and schizophrenia (SCZ) (De Rubeis et al., 2014; International Schizophrenia Consortium, 2008; Kalscheuer et al., 2008; Stefansson et al., 2008)—and initially related to developmental biology, is part of a complex network of proteins communicating with each other at the cellular level. TCF7L2 is a transcriptional regulator of the canonical Wnt‐signaling pathway, a major transducer of β‐catenin activity. It also regulates cell fate specification during development and cell proliferation (Clevers, 2006; Köttgen et al., 2008; MacDonald et al., 2009; Peifer & Polakis, 2000). Furthermore, TCF7L2 may stimulate pancreatic β‐cells proliferation and affect the production of glucagon‐like peptide‐1 (GLP‐1) in intestinal endocrine cells (Jin & Liu, 2008). The Wnt‐signaling pathway controls cell proliferation, stem cell maintenance, cell fate decisions, organized cell movements, and tissue polarity. Within the canonical Wnt‐signaling pathway, the main known signaling factors are the following: low‐density lipoprotein receptor‐related protein 5/6 (LRP5/6), Frizzled receptor (Fz), disheveled (Dvl), β‐catenin, glycogen synthase kinase 3 (GSK3), casein kinase 1 (CK1), axin, adenomatous polyposis coli (APC), and DNA‐bound T cell factor/lymphoid enhancer factor (TCF/LEF) (van Amerongen & Nusse, 2009). In 1999, variants in the *TCF7L2* gene were initially associated with colon cancer (CC) (Duval et al., 1999), and in 2006 with type 2 diabetes mellitus (T2D) (Grant et al., 2006). The *TCF7L2* single nucleotide polymorphisms (SNPs) rs7903146 and rs12255372, namely alleles rs7903146‐T and rs12255372‐T, confer the greatest odds ratio (OR) ~1.4 for T2D‐risk (Grant et al., 2006). As *TCF7L2* is the gene predisposing to the greatest T2D‐relative risk, it predicts, in individuals with impaired glucose tolerance, conversion to T2D (Florez et al., 2006; Lyssenko et al., 2007).

The association of *TCF7L2* with T2D is one of the most powerful genetically discoveries in studies of complex diseases, as it has been consistently replicated in multiple populations with diverse genetic origins (Del Bosque‐Plata et al., 2021). TCF7L2 and other proteins of the Wnt network have been related to various human disorders, reflecting their role in their pathogenesis (Lucero et al., 2010).

This review describes what is known to date about the *TCF7L2* T2D‐variants association in human diseases other than T2D.

### 1.1 Statistical measures

We report ORs and *p* values (p) related to the data. The OR is a measure of association between an exposure (or a genetic variant) and an outcome (or a disease). The OR represents the odds that a disease will occur given a particular gene variant under study, compared to the odds of the disease occurring in the absence of that genetic variant. For OR = 1, the variant does not affect odds of disease; for OR > 1, the variant is associated with higher odds of disease; and for OR < 1, the variant is associated with lower odds of disease.

P is the level of significance of a statistical test, representing the probability of the occurrence of a given event. In all analyses, a *p *< 0.05 was considered statistically significant, meaning that the probability that the data are due to random choice is <.05.

The *p*(trend) reflects the trend) of *p*, thus representing the probability that the data reflect a random result and not a true association.

## 2 *TCF7L2*‐T2D VARIANTS AND MISSION, BEYOND T2D

T2D is the most common form of diabetes, characterized by increased glycemia contributed by impaired insulin action or insulin‐resistance (IR), and beta‐cell dysfunction, and it is associated with obesity (Carlsson, 2019).

In Table 1, we show the role of the T2D‐risk variants studied in diverse pathologies (Table 1), and in Table 2, we show the most common *TCF7L2* variants with pleiotropic effects in human diseases (Table 2).

**Table 1:** *TCF7L2* variants associations in human diseases

Disease	*TCF7L2*‐SNPs	Description
Type 2 diabetes (T2D)	rs7903146	Main variants increasing the risk of T2D (Del Bosque‐Plata et al., 2021)
	rs12255372	
	rs7901695	
	rs7895340	
	rs11196205	
	rs11196218	
	rs290487	
	rs114770437	
	rs7896811	
	rs11196199	
Type 1 diabetes (T1D)	rs7903146	The variant increases T1D risk, independently from overweight status, since it is linked to higher risk of autoimmunity (Leslie & Grant, 2018; Redondo et al., 2014; Redondo, Geyer, et al., 2018; Redondo, Steck, et al., 2018)
Gestational diabetes mellitus (GDM)	rs7903146	rs7903146‐TT increases the risk of GMD fivefold (Franzago et al., 2017)
Latent autoimmune diabetes (LADA)	rs12255372, rs7903146	The effect size of *TCF7L2* variants is similar for LADA and T2D (Cervin et al., 2008; Lukacs et al., 2012; Szepietowska et al., 2010; Zampetti et al., 2010)
Obesity	rs12255372, rs7903146	*TCF7L2* variants do not have a direct relationship with adiposity but influence glycemic indexes, increasing T2D risk in subjects with and without overweight condition (Kimura et al., 2018; Vazquez‐Roque et al., 2011; Wrzosek et al., 2019)
Metabolic syndrome (MetS)	rs12255372, rs7903146	rs7903146 increases the risk of hypertension in patients with T2D; *TCF7L2* variants increase glycemic indexes and T2D risk in MetS patients (Katsoulis et al., 2018; Phillips et al., 2012; Rattanatham et al., 2017)
Diabetic nephropathy	rs7903146, rs7901695	*TCF7L2* variants are significantly associated with CKD progression (Franceschini et al., 2012; Köttgen et al., 2008)
Small‐bowel Crohn's disease (CD)	rs3814570, rs10885394, rs10885395	*TCF7L2* variants contribute to deficient defensins production in the intestine epithelium (Koslowski et al., 2009)
Cancer	rs12255372, rs7903146, rs6983267	*TCF7L2* variants are associated with: gastric, colon and rectal cancer; lung cancer; breast and prostate cancer; and renal cancer (Agalliu et al., 2008; Folsom et al., 2008; Kasper et al., 2009; Lee et al., 2016; Meyer et al., 2010; Naidu et al., 2012; Sainz et al., 2012; Torres et al., 2016; Tuupanen et al., 2009; Zhao et al., 2010)
Schizophrenia	rs7903146, rs12573128, rs1033772	*TCF7L2* variants are significantly associated with schizoid disorders (Alkelai et al., 2012; Hansen et al., 2011; Irvin et al., 2009)
Bipolar disorder	rs290484, rs413267, rs290475, rs10787476, rs1885510, rs6585209, rs7895307	Seven *TCF7L2* variants are significantly associated with elevated BMI related to maniac depressive disorder (Cuellar‐Barboza et al., 2016; Winham et al., 2014)
Cystic fibrosis (CF)	rs7903146	rs7903146 increases nearly threefold T2D risk in patients with CF, and anticipates, particularly in men, symptom onset time of almost 7 years (Blackman, Hsu, Ritter, et al., 2009; Blackman, Hsu, Vanscoy, et al., 2009)
Premature adrenarche	rs7903146	rs7903146 could be related to the premature appearance of secondary sexual characteristics (Lappalainen et al., 2009)
Polycystic ovarian syndrome (PCOS)	rs7903146, rs11196236, rs11196229	Different *TCF7L2* polymorphisms may be associated with PCOS (Biyasheva et al., 2009)
Cardiovascular disease	rs12255372, rs7903146	*TCF7L2* variants are significantly associated with cardiovascular disease (Boccardi et al., 2010; Kucharska‐Newton et al., 2010; Prokunina‐Olsson & Hall, 2010)

Abbreviations: BMI, body mass index; CKD, chronic kidney disease; T2D, type 2 diabetes.

**Table 2:** Most common *TCF7L2* variants with pleiotropic effects in human diseases

*TCF7L2*‐SNPs	Condition
rs7903146	T2D; T1D; GDM; LADA; it influences glycemic indexes, increasing T2D risk in subjects with and without overweight condition; it increases the risk of hypertension in patients with T2D; diabetic nephropathy; cancer; schizophrenia; CF; premature adrenarche; PCOS; cardiovascular disease
rs12255372	T2D; LADA; it influences glycemic indexes, increasing T2D risk in subjects with and without overweight condition; it increases glycemic indexes and T2D risk in MetS patients; cancer; cardiovascular disease
rs7901695	T2D; diabetic nephropathy

Abbreviations: CF, cystic fibrosis; GMD, gestational diabetes mellitus; LADA, latent autoimmune diabetes in adults; MetS, metabolic syndrome; PCOS, polycystic ovarian síndrome; T1D, type 1 diabetes; T2D, type 2 diabetes.

### 2.1 Type 1 diabetes (T1D)

T1D, once known as juvenile diabetes or insulin‐dependent diabetes, is a chronic condition in which the pancreas produces little or no insulin, a hormone that is necessary for survival because it allows glucose to enter cells to produce energy (WHO, 2020).

T1D presents phenotypic diversity that signifies a heterogeneous etiopathogenesis. Within T1D, several researchers have explored the role of *TCF7L2* variants associated with T2D. Recently, in a study of 71 T1D‐children, 21.1% had a sole antipancreatic islet antibody (*N* = 15) and showed a *TCF7L2* rs7903146‐genotype distribution of 40% CC, 26.7% CT, and 33.3% TT, while T1D‐children with ≥2 islet autoantibody showed a genotype distribution of 50% CC, 42.9% CT, and 7.1% TT (*p* = 0.024). Additionally, children with a sole autoantibody had characteristics reflecting milder autoimmune β‐cell destruction compared to children with ≥2 autoantibodies. Among lean children (body mass index [BMI] < 85th percentile; *N* = 36), 45.5% of those with a single autoantibody were rs7903146‐TT homozygotic compared with 0% of those with ≥2 autoantibodies (*p* < 0.0001). These results suggest that, in children with only mild islet autoimmunity, mechanisms associated with *TCF7L2* genetic variation contribute to the development of T1D, and this contribution is greater in the absence of obesity. It is plausible to think that the rs7903146‐TT variant is most deleterious in the perinatal stage, thus reducing in carriers the chance of surviving pregnancy, childbirth, and lactation (Redondo et al., 2014).

To know if *TCF7L2* locus influences progression of T1D‐islet autoimmunity, Redondo et al. evaluated in relatives of T1D‐patients single to multiple (≥2) autoantibody positivity (Redondo et al., 2018). During follow‐up with 244 participants of the T1D‐TrialNet Pathway to Prevention study (median: 5.2 years, range = 0.2–12.6), 62% of the single autoantibody‐positive subjects developed multiple autoantibody positivity. Among single glutamic‐acid decarboxylase 65 (GAD65) autoantibody‐positive participants (*N* = 158), those who carried ≥1 *TCF7L2*‐risk allele(s) had a lower progression rate to multiple autoantibody positivity (hazard ratio [HR] 0.65, *p* = 0.033) than those who did not, after adjusting for age and human leukocyte antigen (HLA) T1D‐risk haplotypes. Among subjects who were either only tyrosine phosphatase‐related islet antigen 2 (IA‐2)‐ or insulin autoantibody‐positive, carrying ≥1 *TCF7L2*‐risk allele(s) was not a significant factor, but in those who were overweight or obese, it increased the progression to multiple autoantibody positivity (HR = 3.02, *p* = 0.016), even with age adjustment (Redondo et al., 2018).

Specifically, *TCF7L2*‐T2D risk allele rs4506565, in linkage disequilibrium (LD) with T2D‐variant rs7903146, reduces the risk of progression to multiple autoantibody positivity among single GAD65 autoantibody‐positive subjects, particularly if T1D‐risk HLA‐haplotypes are absent. In subjects with single microinsulin autoantibody (mIAA) or IA‐2 autoantibody, the rs7903146‐T risk allele interacts with obesity or overweight status to mark faster progression to multiple autoantibody positivity. Thus, the T2D‐associated *TCF7L2* locus influences progression of islet autoimmunity, with differential effects given by autoantibody specificity and interaction of obesity or overweight status. The findings suggest a role of the *TCF7L2*‐locus in islet autoimmunity in subsets of individuals and support an interrelationship between genetic, immunologic, and metabolic factors in T1D pathogenesis (Redondo et al., 2018).

Larger studies are required to define whether the genetic sensitivity of islet β‐cells to immunologic or metabolic stress contributes to the risk of developing diabetes, irrespective of its type (Leslie & Grant, 2018).

### 2.2 Gestational diabetes mellitus (GDM)

GDM is defined as any degree of glucose intolerance that starts, or that is first recognized, during pregnancy. The definition applies whether insulin or only diet modification is used for treatment and whether or not the condition persists after pregnancy (Franz et al., 1994).

*TCF7L2* is associated with susceptibility to GDM independently of the T1D‐risk HLA‐DQB1*0602 allele, autoantibodies to pancreatic β‐cells, and other factors (e.g., maternal age, number of pregnancies, familial history of diabetes, and other genotypes).

In 168 pregnant Caucasian women, the rs7903146‐TT genotype was associated with a more than fivefold increased GDM‐risk (OR = 5.4; 95% CI = 1.5–19.3) (Franzago et al., 2017). The same but extended cohort (104 women with GDM and 124 pregnant control subjects) showed under a codominant model that the TT genotype is significantly more frequent among GDM patients than healthy controls (26.9% vs. 13.7%; OR = 2.56; 95% CI: 1.24–5.29, *p* = 0.04) (Franzago et al., 2018). The rs7903146‐TT variant modulates pancreatic β‐cell function and is one of the most important T2D‐ and GDM‐susceptibility variants across different populations.

### 2.3 Latent autoimmune diabetes in adults (LADA)

The World Health Organization classifies LADA as a subtype of slowly progressing T1D, although it frequently presents clinical features similar to those of T2D. LADA shares genetic characteristics of T1D: *HLA*, insulin gene [*INS*], variable number tandem repeat [*VNTR*], and nonreceptor type 22 gene [*PTPN22]*. However, the HLA‐complex genes contribute to approximately half of the T1D‐genetic susceptibility (Noble et al., 1996). Although T2D‐genetic risk is attributed to many common genetic variants contributing a small amount to T2D heritability (Fuchsberger et al., 2016), LADA‐patients carry an increased TT‐genotype frequency at the *TCF7L2*‐T2D rs7903146 variant compared to controls. (15% vs. 6%; *p* = 0.03) (Cervin et al., 2008; Szepietowska et al., 2010). A meta‐analysis demonstrated that the rs7903146‐T variant is a population‐independent susceptibility locus for LADA in Europeans. The effect size is similar for LADA and T2D. The genetic effect on the risk for LADA may be modulated by BMI; the lower the BMI, the greater the genetic effect (Lukacs et al., 2012). In LADA patients, *TCF7L2*‐T2D common variants rs12255372 and rs7903146 are associated only with low Glutamic Acid Decarboxylase Antibody (GADA) titers, showing heterogeneous genetic and clinical characteristics in LADA patients according to GADA titers; the authors hypothesize that the *TCF7L2*‐risk alleles predispose to lower insulin secretion in these patients (Zampetti et al., 2010).

## 3 OBESITY

Overweight conditions and obesity are defined as abnormal or excessive fat accumulation that presents a risk to health. A BMI ≥ 25 is considered overweight, and ≥ 30 is obese (WHO, 2020). Obesity is determined by a complex interaction of environmental factors with genetic variants implicated in pathways of glucose‐homeostasis regulation, thereby potentially modulating T2D development in obese subjects (Kahn, 2003; Mokdad et al., 2003). Obese carriers of the TCF7L2‐rs7903146 TT‐genotype have 2.62‐times higher odds of T2D compared to those with other genotypes, and this relationship appears even stronger after adjusting for obesity‐onset age, male gender, dyslipidemia, and lower serum total adiponectin (Wrzosek et al., 2019). In addition, the risk of developing impaired fasting glycemia associated with the TT‐genotype is stronger in obese than nonobese Caucasians (Yan et al., 2010).

Significantly, the rs7903146‐T variant has been associated with gastric functional alterations potentially predisposing to obesity (e.g., reduction of fasting gastric volume and rapid gastric emptying) (Vazquez‐Roque et al., 2011). In contrast, rs7930146‐T has been associated with T2D in obese Polish adults.

Furthermore, a study in Mexican children showed that the rs12255372‐T variant protects them from obesity, although replication is needed (Klünder‐Klünder et al., 2011). Vascular GLP‐1 receptor, a target of T2D‐therapy, and *TCF7L2* expression were significantly downregulated in obese subjects, indicating that *TCF7L2* modulates vascular GLP‐1 receptor expression (Kimura et al., 2018).

## 4 METABOLIC SYNDROME (MetS)

MetS is a heterogeneous disorder defined by the presence of at least three of the following traits: waist circumference >40 inches in men or >35 inches in women, fasting glycemia >100 mg/dl or therapy, blood pressure >130 mmHg systolic or 85 mmHg diastolic or therapy, and triglyceridemia >150 mg/dl or therapy, high‐density lipoprotein <40 mg/dl in men or <50 mg/dl in women or therapy (Grundy et al., 2005).

The accumulation of the various traits in an individual raises the risk of developing atherosclerotic cardiovascular disease (CVD), IR, diabetes mellitus, and vascular and neurological complications, such as a cerebrovascular accident (van der Pal et al., 2018).

*TCF7L2* rs7903146‐T influences MetS‐risk, which increases with female gender and saturated fatty‐acids intake (Phillips et al., 2012). Also, rs7903146‐T and rs12255372‐T variants play a key role in T2D‐onset among individuals with MetS. In fact, subjects with MetS who develop T2D have a genetic predisposition to β‐cell dysfunction: the rs12255372‐T variant was more frequent in patients with MetS and impaired glucose metabolism (IGM, 48.3%) than in those with MetS and normoglycemia (NGM, 19.4%, *p* <  0.001); and the rs7903146‐T allele was more frequent in patients with MetS and IGM (44.6%) compared to subjects with MetS and NGM (18.1%, *p* <  0.001). The presence of both variants in MetS is a powerful predictor of glucose impairment, increasing the risk more than fourfold (Katsoulis et al., 2018). Furthermore, it has been found that the variant rs7903146 increases the risk of hypertension in patients with T2D (Rattanatham et al., 2017).

## 5 DIABETIC NEPHROPATHY (DN)

Diabetic nephropathy, also known as diabetic kidney disease (Kittell, 2012), is the chronic loss of kidney function occurring in those with diabetes mellitus. DN is one of the leading causes of chronic kidney disease (CKD) and end‐stage renal disease (ESRD) (Longo et al., 2012). It has been classically defined by the presence of proteinuria >0.5 g/24 h (Mogensen, 1984). It affects approximately 40% of T1D and T2D patients and increases the risk of death, mainly from cardiovascular causes. It is currently defined by increased urinary albumin excretion (UAE) in the absence of other renal diseases and is subdivided into microalbuminuria (UAE > 20 μg/min and ≤199 μg/min) and macroalbuminuria (UAE ≥ 200 μg/min). Genetic risk, increased blood pressure, and hyperglycemia are the main risk factors for DN; however, smoking, dyslipidemia, and dietary protein amount may also increase risk (Gross et al., 2005).

The role of *TCF7L2* in CKD progression has been tested in several population‐based studies, including the Atherosclerosis Risk in Communities Study (ARIC; *N* = 11,061 self‐identified whites and *N* = 4,014 blacks). The T2D‐risk alleles at rs7903146 and rs7901695 were significantly associated with CKD progression among ARIC participants and those without baseline T2D (Köttgen et al., 2008). In American Indians with early‐onset DN, *TCF7L2* variants have been associated with lower estimated glomerular filtration rate, but not with urinary albumin‐to‐creatinine ratio (Franceschini et al., 2012).

In 1,065 patients with ESRD and 924 healthy controls, the rs7903146‐T variant was significantly associated with ESRD. This result aligns with previous findings of *TCF7L2*‐variant association with renal function and CKD progression (Buraczynska et al., 2014).

## 6 SMALL‐BOWEL CROHN'S DISEASE (CD)

Small‐bowel CD is a form of inflammatory intestinal disease that implies chronic inflammation of the gastrointestinal tract. It generally affects the intestines and can present from the mouth to the rectum; it is characterized by a reduced expression of the antimicrobial α‐defensins produced by Paneth cells, the principal cell type of the small intestine epithelium, along with goblet cells, enterocytes, and enteroendocrine cells. TCF7L2 directs the differentiation of Paneth cells and directly regulates α‐defensins type 5 (HD5) and 6 (HD6) expression, which is associated with the levels of these antimicrobial peptides. To investigate the possible association of ileal CD with *TCF7L2*, 2.1 kb of region 5ʹ of the gene was sequenced in patients with ileal CD and controls (*N* = 10 per each group). Eight variants were identified, of which three (rs3814570, rs10885394, and rs10885395) are in LD and more frequent in CD patients; rs3814570 is localized in a potential regulatory region. A study including 1,399 healthy individuals, 225 patients with colonic CD, 784 patients with ileal CD, and 785 patients with ulcerative colitis demonstrated the association of the rs3814570 variant with ileal CD (OR = 1.32; 95% CI = 1.08–1.62; *p* = 0.00686), and found no association with colonic CD or ulcerative colitis; within ileal CD, association with constipation was also present (OR = 1.38; 95% CI = 1.03–1.84; *p* = .02882). The *TCF7L2* association with ileal CD indicates that TCF7L2 plays a role in Paneth‐cell defensin disease as a primary factor in CD pathogenesis (Koslowski et al., 2009).

## 7 CANCER

Cancer is a large group of diseases that can start in almost any organ or tissue of the body when abnormal cells grow uncontrollably, go beyond their usual boundaries to invade adjoining parts of the body, and/or spread to other organs (WHO, 2020). As *TCF7L2* gene variation may be involved in T2D development and in different mechanisms of other pathologies, such as those related to hyperglycemia, the pathophysiological mechanisms‐related pathways are in turn strongly linked to cancer etiology. In particular, within the Wnt/β‐catenin signaling pathway, we can highlight impaired mechanisms of regulation of the cell cycle, cell differentiation, apoptosis, and response to oxidative stress, and low function of gluconeogenesis (Geoghegan et al., 2019; Oh et al., 2012; Silbernagel et al., 2011).

In the prospective ARIC‐cohort study (13,117 middle‐aged adults, cancer‐free in the years 1987–1989), the rs7903146‐T allele was analyzed in relation to various common cancers (Folsom et al., 2008). In 2000, T2D was found to have an inverse marginal incidence with prostate cancer; no association was found between T2D and the incidence of colorectal cancer (CRC), CC, cancer of the lung, or of the breast (Folsom et al., 2008). The rs7903146‐T allele is associated with lung cancer in Caucasians but not in African‐Americans. Furthermore, the rs7903146‐T allele, presenting with a frequency of 30% in Caucasians, is associated with the risk of CC in the general population (approximately 17%). This association with lung cancer appears due to an independent genetic effect that is not explained by the presence of T2D (Folsom et al., 2008).

TCF7L2 has been shown to be a part of a super‐enhancer (SE) called the EphA2‐SE; SEs are large clusters of active transcription enhancers, which promote the expression of critical genes that define cell identity in health and disease. EphA2‐SE deletion, using CRISPR/Cas9, significantly downregulates its target gene EphA2 (Cui et al., 2021). By integrating RNA‐seq data, functional in vitro experiments, and xenograft animal model, Cut et al. discovered that EphA2‐SE plays an oncogenic role and contributes to tumor progression in several tumors, as it recruits FOSL2 and TCF7L2 to drive the expression of the oncogene EphA2 (Cui et al., 2021). EphA2 kinase may have opposite roles in regulating chemotactic cell migration: it mediates ligand‐dependent inhibition and ligand‐independent promotion of cell migration and invasion (Miao et al., 2009). Previous reports have suggested that large cancer‐associated enhancer clusters or SE may be particularly sensitive to perturbations, making them putative therapeutic targets (Bradner et al., 2017).

### 7.1 Gastric cancer (GC)

In a study of 129 GC‐ biopsies from Venezuelan patients, the *TCF7L2* rs7903146 TT genotype was associated under the recessive model with GC‐ risk (OR = 3.11; 95% CI: 1.22–7.92; *p* = 0.017) and poorly differentiated GC (OR = 3.65; 95% CI: 1.25–10.62; *p* = 0.018), and the rs7903146 T allele conferred a significantly increased risk of moderate/well‐differentiated GC under the dominant model (OR = 2.55; 95% CI: 1.35–4.80; *p* = 0.004); these findings should be replicated in a larger data set. No association was detected between rs12255372 and GC‐ risk (Torres et al., 2016).

### 7.2 CRC and CC

German T2D‐patients carrying the rs7903146‐T allele had an increased risk of CRC (*p*(trend) = 0.02). Further analysis revealed gender‐specific effects: the rs7903146‐T allele was associated with an increased risk of CRC only in women (*p*(trend) = 0.003) (Sainz et al., 2012). β‐Catenin accumulation, and its mediated transactivation of certain TCF7L2‐target genes, plays a key role in colorectal carcinogenesis (Shitashige et al., 2007). Another study did not confirm rs7903146‐T association with CC (1578 cases, 1966 controls), but did find a statistical interaction between the variant and the recent use of aspirin and/or nonsteroidal anti‐inflammatory drugs (NSAIDs), suggesting that CC risk associated with rs7903146‐T is mediated by the use of aspirin and/or NSAIDs (Freedman et al., 2009). There is evidence that CC predisposition is associated with an enhancer element containing the *TCF7L2*‐rs6983267 variant, whose homozygosity increases CC risk circa 1.5 times, and the G allele increases TCF7L2 transcription factor binding in vivo and in vitro. Complete genomic chromatin immunoprecipitation (ChIP) assay reveals this element as the strongest TCF7L2‐binding site (Tuupanen et al., 2009). By a genomic binding profile using the CC cell line LS174T, it was identified that TCF7L2 binds to 6,868 genomic sites. In the human intestine, the majority of TCF7L2‐transcriptional targets present on the same *TCF7L2*‐DNA molecule (called “target in cys”), and not in a DNA molecule on which TCF7L2 is acting (called “targets in trans”), have been defined by combining genetic information with expression data. The motif‐search algorithm defines the TCF7L2‐binding site as an evolution‐conserved AC/GA/TTCAAAG motif, although 30% of the binding regions do not contain a typical binding motif, which could be due to factors such as the presence of nonidentified atypical motifs, protein‐protein interactions with other factors directly recruited to those regions, or to the fact that some *TCF7L2*‐association signals reflect association at other sites (Hatzis et al., 2008).

By using the CRC line HCT116, ChIP, and sequencing, a total of 1,095 discrete sites binding to TCF7L2 were found in the genome, and a subgroup of 548 sites was found within 5 kb of sequences annotated in the National Center for Biotechnology Information Reference Sequences. Analysis of the metabolic pathways involved in these binding sites demonstrated that the most representative functions were related to T2D, coronary artery disease, and other significant disease risk loci, of which most were primarily represented by metabolic and cardiovascular traits, and a few were related to cancer and inflammatory and CVDs. The most enriched specific traits were T2D and height (Kasper et al., 2009; Zhao et al., 2010).

### 7.3 Prostate cancer

Various studies and meta‐analyses report that patients with T2D have a lower risk of developing prostate cancer. Even if the concentration of sex hormones and the expression of sex hormone receptors are among the main risk factors for a higher incidence of prostate cancer, carrying the *TCF7L2* rs12255372‐T allele is associated with prostate cancer progression and metastases. Also, studies show that the risk of death from prostate cancer increased in subjects with T2D compared to those without T2D (Kasper et al., 2009; Lee et al., 2016; Lucero et al., 2010; Meyer et al., 2010).

Men who are homozygotic for the *TCF7L2*‐rs12255372 T allele have a high relative risk for more aggressive prostate cancer, defined by a high Gleason score (OR = 1.7; 95% CI = 1.0–2.8) or by a high regional and/or distal stage (OR = 1.7; 95% CI = 1.1–2.6) (Agalliu et al., 2008).

### 7.4 Breast cancer

A study with 387 patients with breast cancer compared to healthy subjects found that the rs7903146‐T allele was significantly associated with lymph node involvement (*p* = 0.003), while the rs12255372‐T allele was not associated with clinicopathological characteristics (Naidu et al., 2012).

### 7.5 Clear‐cell renal cell carcinoma (CCRCC)

Silencing *TCF7L2* using miRNA in CCRCC lines causes cell sensitivity at a clinically relevant X‐ray dose, and the effect is restricted to tumor cells with high TCF7L2 activity. This phenomenon is thought to be independent of β‐catenin. Radiosensitization is the consequence of dysregulation of TCF7L2/Wnt‐target genes, the arrest of the G(2)/M‐induced silencing phase, and postradiation damage in cell cycle progression. This new mechanism, which measures both chemo‐ and radio‐resistance, suggests that TCF7L2 could be a molecular target for sensitizing radiotherapy‐resistant tumor cells (Kendziorra et al., 2011). Kojima et al. have made integrated analyses of copy number variants and gene expression data to identify metastasis‐related genes in CCRCC. They found that both TCF7L2 and FOXO1, a key downstream mediator in the PI3K/AKT pathway, are involved in the acquisition of the malignant phenotype in CCRCC (Kojima et al., 2010).

## 8 PSYCHIATRIC DISORDERS

A psychiatric disorder is a mental illness that greatly disturbs an individual's thinking, moods, and/or behavior and seriously increases the risk of disability, pain, death, or loss of freedom.

### 8.1 Schizophrenia (SCZ)

SCZ is characterized by distortions in thinking, perception, emotions, language, sense of self, and behavior. Common experiences include hallucinations (hearing voices or seeing things that are not there) and delusions (fixed, false beliefs) (WHO, 2020).

Exposure to antipsychotics induces metabolic dysregulation in some patients; however, SCZ is *per se* associated with increased T2D‐risk in drug‐naïve patients (Fernandez‐Egea et al., 2009). In African‐American patients with psycho‐affective or schizophrenic disorders, the *TCF7L2* rs7903146‐T variant was associated with T2D (OR = 1.4; *p* = 0.03) by the additive model, and more so by the recessive model (OR = 2.4; *p* = 0.004). Of interest, marginal significance was found for interaction between rs7903146‐T and antipsychotic treatment (Irvin et al., 2009).

Alkelali et al. reported linkage of the 10q24‐q26 region to SCZ in Arab‐Israeli families with multiple SCZ‐affected individuals (Alkelai et al., 2012). Within the identified locus, they found significant SCZ association with the *TCF7L2*‐rs12573128 intronic SNP (*p*  = 7.01 × 10⁻^6^) and the nearby rs1033772‐intergenic SNP (*p*  = 6.59 × 10⁻^6^). These results independently support previous findings regarding a possible role of *TCF7L2* in SCZ susceptibility (Alkelai et al., 2012). In the Danish population, the rs7903146‐T allele was associated with SCZ in the discovery sample (*p* = 0.0052) and in the replication sample (OR = 1.07; 95% CI = 1.01–1.14; *p* = 0.033) (Hansen et al., 2011). In 191 elderly T2D‐patients, four *TCF7L2* SNPs (rs7901695, rs7903146, rs11196205, and rs12255372) were tested for association with volumes of white matter hyperintensities, gray matter, amygdala, and hippocampal imaging by using brain magnetic resonance. *TCF7L2* polymorphisms were found to be associated with a lower volume of the amygdala. These findings should be confirmed in other populations and expanded in other regions of the limbic system. Carriers of these *TCF7L2* polymorphisms may be predisposed to increased risk for schizoid disorders (Ganmore et al., 2019).

### 8.2 Bipolar disorder (BD)

BD is a serious mental illness in which common emotions become intensely and often unpredictably magnified; individuals with BD can quickly swing from extremes of happiness, energy, and clarity to sadness, fatigue, and confusion (WHO, 2020).

BD is a complex disease associated with several hereditary traits, including a higher BMI. Although overall there was no evidence for *TCF7L2* SNP‐marginal effects on BD, BMI modified the association of the rs12772424‐T variant with BD (Winham et al., 2014). The *TCF7L2*–BMI interaction was significant at the gene level (*p* = 0.042), with 7 of the 26 tested *TCF7L2‐*SNPs showing SNP‐BMI interaction effects (*p* < 0.05). The strongest evidence of *TCF7L2‐*SNP and BMI interaction in BD was observed for rs7895307 (*p* = 0.006). *TCF7L2* expression showed significant enrichment of association with the expression of other genes in the Wnt canonical pathway (Cuellar‐Barboza et al., 2016).

## 9 CYSTIC FIBROSIS (CF)

CF is a chronic lung disease causing accumulation of thick and sticky mucus in the lungs, altering the pancreas and contributing to CF‐secondary diabetes, and impairing other organs. In CF, the rs7903146‐T variant confers a nearly threefold T2Drisk (HR = 1.75 per allele; 95% CI = 1.3–2.4; *p* = 0.0006) and diminishes the mean age of its diagnosis by 7 years. In CF patients without glucocorticoid treatment, the effect is even greater (HR = 2.9 per allele; 95% CI = 1.7–4.9; *p* = 0.00011). Therefore, the rs7903146‐T variant is a risk modifier for CF‐secondary diabetes (Blackman, Hsu, Ritter, et al., 2009; Blackman, Hsu, Vanscoy, et al., 2009).

## 10 ENDOCRINE DISORDERS

Endocrine disorders are diseases related to the hormone‐producing glands of the body. These disorders can affect multiple parts of the body and have a wide range of signs and symptoms, depending upon the gland being affected (Kronenberg et al., 2016).

### 10.1 Premature adrenarche (PA)

PA, also referred to as premature pubarche, refers to the early appearance of pubic hair, axillary hair, or both in children without other signs of puberty (Voutilainen & Jääskeläinen, 2015).

The rs7903146‐T allele is more frequent in PA subjects with low height and weight than in controls, suggesting that this allele may be involved in the pathogenesis of PA in lean subjects (Lappalainen et al., 2009).

### 10.2 Polycystic ovarian syndrome (PCOS)

PCOS, a common endocrine disorder characterized mainly by irregular menses and hyperandrogenism, is frequently associated with IR and obesity. The role of IR and obesity in this syndrome is not clear; thus, the role of obesity and T2D‐variants has been investigated in PCOS and its specific traits (Rojas et al., 2014).

In an association study with 386 PCOS patients (defined by the Rotterdam criteria) and 1,971 control women, the rs7903146‐T variant was strongly associated with body weight (Biyasheva et al., 2009). Furthermore, *TCF7L2* SNPs were tested in PCOS subjects for association with PCOS quantitative traits (e.g., BMI, total testosterone, dehydroepiandrosterone sulfate [DHEA‐S], sex hormone‐binding globulin [SHBG], proinsulin:insulin ratio [a marker of pancreatic β‐cell dysfunction]). *TCF7L2‐*PCOS association was found within 100 kb of the Caucasian T2D‐locus, which, after multiple corrections and population stratification, harbored one significant SNP (rs11196236‐G, *p*_observed _= 9.0 × 10^−4^, *p*_corrected _= 0.047). The proinsulin:insulin ratio with significant PCOS association (rs4506565, *p*_observed_ = 2.1 × 10^−4^, *p*_corrected_ = 0.010) maps to the Caucasian *TCF7L2* T2D‐locus. Other PCOS‐metabolic measures were nominally associated without holding multiple corrections (e.g., BMI, SHBG, fasting insulinemia, waist circumference). Within the PCOS locus, testosterone and DHEA‐S levels were nominally associated (rs37500805 and rs6585206, respectively). Furthermore, rs4506565 was robustly associated with PCOS‐glucose intolerance (*p*_observed_ = 9.8 ×10^−5^, *p*_corrected_ = 1.7 × 10^−4^,* N* = 139). The T2D locus and the PCOS locus are not in LD and are inherited independently and associated with different biochemical features of PCOS (Biyasheva et al., 2009).

## 11 CARDIOVASCULAR DISEASES (CVD)

CVDs are a group of disorders of the heart and blood vessels, including coronary heart disease and cerebrovascular disease. Four out of five CVD deaths are due to heart attacks and strokes, and a third of these deaths occur prematurely in people under 70 years of age (WHO, 2020).

The rs12255372‐TT and rs7903146‐TT genotypes are associated with reduced insulin sensitivity and high cardiosympathetic activity. These effects are independent of GLP‐1 and insulinemia, which suggests the potential role of these variants in increased cardiovascular risk through the augmentation of central nervous system (CNS)‐sympathetic activity (Boccardi et al., 2010). The splicing form of *TCF7L2*‐NE, a neuroendocrine isoform in CNS, has been reported as significantly correlated to the expression of the gene for cocaine‐ and amphetamine‐regulated transcript (CART) (*r* = 0.85, *p* = 0.00046). CART is an important neurotransmitter in the CNS and is involved in diverse fundamental metabolic processes. The functional relationship between *TCF7L2*‐NE and CART is unclear (Prokunina‐Olsson & Hall, 2010).

It has also been reported that body mass modifies the association of the rs7903146‐T allele with incidental coronary heart disease risk (Kucharska‐Newton et al., 2010; Zhao et al., 2010).

## 12 CONCLUSIONS

Although the role of the *TCF7L2* T2D‐variants has been extensively studied in a number of diseases, this has opened the flood‐gates for an entirely new series of challenging questions.

Common polymorphisms of the *TCF7L2* gene have been related to diverse diseases. However, the role of rare mutations in the *TCF7L2* gene in different diseases and diverse ethnic groups has not been explored; thus, larger cohort studies are needed to elucidate their role. Previous findings suggest that genetic variation in different regions of the *TCF7L2* gene is associated with distinct phenotypes, but the pathophysiological mechanisms‐related pathways are not well known.

As ethnic variation in allele frequencies has been found throughout the genome, it is of great importance to study its genetic diversity in different races, ethnicities, and populations to reveal evolutionary patterns and identify variants that contribute to the causes of common diseases.

Within the Wnt pathway, TCFs function can be inhibitory and activating dependent on the context; more research is needed in this area. In particular, dissecting the *TCF7L2* functional regions and understanding their impact on specific phenotypes will be important to comprehend their role in diverse human diseases and highlight a possible pleiotropic role in human comorbid disorders.

## 13 PERSPECTIVES

Fifteen years ago, intronic variants in *TCF7L2* were associated with T2D; since then, a huge amount of knowledge has been accumulated in diverse contexts, opening up enormous possibilities for follow‐up. Among the scientific opportunities to explore are the mechanisms over which TCF7L2 exerts its effect on diverse diseases, which may vary depending on the context of the specific cell or tissue. A deeper knowledge of the *TCF7L2* variants and their potential differential cell‐ or tissue‐specific role along with a deeper knowledge of a more precise role of the gene variation in diverse races and ethnicities, as well as an understanding of the role of variation in natural selection, will lead us to a more thorough understanding of the gene and its variants effects in disease.

The Wnt/β‐catenin signaling is subject to regulation at different levels, and its effects vary by cellular, temporal, and spatial contexts. Thus, it is difficult to generalize whether any end pathway results in activation or inhibition. A clear example is within cancer, where, if oversimplified, the Wnt/β‐catenin signaling and TCF7L2 pathways can be considered oncogenic or tumor‐suppressive.

We propose that research must be carried out to understand the TCF7L2 molecular mechanisms controlling both localized and temporal expression of their signaling components in the specific cells and tissues affected in different diseases. It is expected that high‐throughput methods will help to resolve many questions and provide essential knowledge regarding Wnt/β‐catenin regulation and its perpetual crosstalk with other key signal transduction pathways. Using the integration of genomic data with different types of “omics" will elucidate how disease variants in *TCF7L2* interact with Wnt/β‐catenin signaling, according to the genomic background of each individual. This will further our understanding of the pathogenic role of the pleiotropic variants.

In addition, to study the role of Wnt/β‐catenin signaling and its interaction with TCF7L2 in different diseases, we need to address how the animal and in vitro models recapitulate what is seen in patients with regard to deregulation of Wnt/β‐catenin signaling. These discoveries should be harmonized especially with information related to Wnt/β‐catenin activation or inhibition in human diseases.
